# Comparative transcriptomics of anal fin pigmentation patterns in cichlid fishes

**DOI:** 10.1186/s12864-016-3046-y

**Published:** 2016-09-06

**Authors:** M. Emília Santos, Laura Baldo, Langyu Gu, Nicolas Boileau, Zuzana Musilova, Walter Salzburger

**Affiliations:** 1 Zoological Institute, University of Basel, Vesalgasse 1, 4051 Basel, Switzerland; 2 Institut de Génomique Fonctionnelle de Lyon, Ecole Normale Supérieure, CNRS UMR 5242, 46 Allée d’Italie, 69364 Lyon, Cedex 07, France; 3 Ecology Department, University of Barcelona, Av. Diagonal, 643, 08028 Barcelona, Spain; 4 Department of Zoology, Faculty of Science, Charles University in Prague, Vinicna 7, 128 44 Prague, Czech Republic

**Keywords:** Pigmentation, Diversity, Egg-spot, Blotches, East African cichlids, Gene expression

## Abstract

**Background:**

Understanding the genetic basis of novel traits is a central topic in evolutionary biology. Two novel pigmentation phenotypes, egg-spots and blotches, emerged during the rapid diversification of East African cichlid fishes. Egg-spots are circular pigmentation markings on the anal fins of hundreds of derived haplochromine cichlids species, whereas blotches are patches of conspicuous anal fin pigmentation with ill-defined boundaries that occur in few species that belong to basal cichlid lineages. Both traits play an important role in the breeding behavior of this group of fishes. Knowledge about the origin, homology and underlying genetics of these pigmentation traits is sparse.

**Results:**

Here, we present a comparative transcriptomic and differential gene expression analysis of egg-spots and blotches. We first conducted an RNA sequencing experiment where we compared egg-spot tissue with the remaining portion of egg-spot-free fin tissue using six individuals of *Astatotilapia burtoni*. We identified 1229 differentially expressed genes between the two tissue types. We then showed that rates of evolution of these genes are higher than average estimated on whole transcriptome data. Using quantitative real-time PCR, we found that 29 out of a subset of 46 differentially expressed genes showed an analogous expression pattern in another haplochromine species’ egg-spots, *Cynotilapia pulpican*, strongly suggesting that these genes are involved in the egg-spot phenotype. Among these are the previously identified egg-spot gene *fhl2a*, two known patterning genes (*hoxC12a* and *bmp3*) as well as other pigmentation related genes such as *asip*. Finally, we analyzed the expression patterns of the same gene subset in two species that feature blotches instead of egg-spots, one haplochromine species (*Pseudocrenilabrus philander*) and one ectodine species (*Callochromis macrops*), revealing that the expression patterns in blotches and egg-spots are rather distinct.

**Conclusions:**

We identified several candidate genes that will serve as an important and useful resource for future research on the emergence and diversification of cichlid fishes’ egg-spots. Only a limited degree of conservation of gene expression patterns was detected between the egg-spots of the derived haplochromines and blotches from ancestral haplochromines, as well as between the two types of blotches, suggesting an independent origin of these traits.

**Electronic supplementary material:**

The online version of this article (doi:10.1186/s12864-016-3046-y) contains supplementary material, which is available to authorized users.

## Background

Animal pigmentation patterns are highly variable phenotypes both at the intra- and inter-specific level, and represent prominent traits to study the genetics of species diversification and adaptation (reviewed in [1–3]). The functionality of color patterns can readily be assessed in most cases, given that these traits often evolve in response to adaptation to the environment via natural selection (e.g. inter- and intra-specific communication, camouflage and mimicry), or co-vary with female choice via sexual selection [4–6]. The outcome of these two types of selection regimes can be different, with the former often producing cryptic phenotypes, where coloration mimics the environment, while the latter generates conspicuous phenotypes, where males typically display bright colors driving female choice or male-male competition [4–6]. Despite the high evolutionary significance of color patterns, the genetic mechanisms underlying their formation and diversification often remain elusive [1–3].

Recent work in fish model systems, especially in zebrafish, has started to uncover the genes and cellular processes involved in pigmentation pattern formation [7–9]. Pigmentation patterns are determined by the specification of different types of neural crest derived pigment cells – the chromatophores [10] – that contain different light absorbing pigments: melanophores contain black eumelanin pigments; erythrophores and xantophores contain yellow-red carotenoid and pteridine pigments; cyanophores contain a blue pigment of unknown composition; and finally, iridophores contain purine crystals that produce metallic iridescence [11]. Differences in the arrangement, position, and density of these cells leads to the diversity of color patterns present in nature. These differences depend on a variety of factors including neural crest cell migration, specification, proliferation, and survival [7–9, 11].

**Fig. 1:**
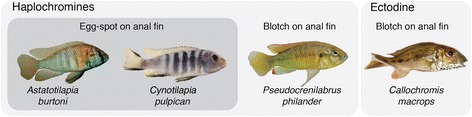
Representative males from the four species analyzed: two haplochromine species displaying egg-spots in their anal fins (*A. burtoni* and *C. pulpican*), a basal haplochromine species (*P. philander*) and an ectodine species (*C. macrops*), both showing orange blotches in their anal fin

In this study, we address the molecular basis of two novel and conspicuous pigmentation traits found in the anal fin of male cichlid fishes – egg-spots and blotches (Fig. 1). Egg-spots represent an evolutionary novelty that emerged only once in the haplochromine lineage, the most species-rich group of East African cichlids [12, 13]. These circular markings consist of a central circular area containing xanthophores and iridophores, surrounded by an outer transparent ring [14, 15]. They are primarily found in males and show an extreme inter- and intra-specific variability in number, color, and position on the fin [13–16]. Egg-spots have been the subject of intense studies suggesting a signaling function in the peculiar mating behavior of the mouth-brooding haplochromines. They are likely sexually selected via female choice in some species [17, 18] and via male-male competition in others [19–21]. Blotches, on the other hand, are patches of conspicuous anal fin pigmentation with ill-defined boundaries and occur only in a handful of cichlid species, including some basal haplochromines [13–15] and ectodine cichlids from Lake Tanganyika (Fig. 1). As with egg-spots, they are mostly found in males and their function might also be linked to courtship behavior, although this has been less extensively studied [12]. The origin and evolutionary trajectory of these anal fin patterns remains unclear. Due to the phylogenetic position of the species showing blotches as sister-group to the egg-spot bearing haplochromines [13–15], it might be speculated that egg-spots are derived from the blotch-pattern, which would make the two phenotypes homologous.

Convergent evolution is widespread in East African cichlid adaptive radiations, not only between lakes [22, 23], but also within a single lake [24]. For example, haplochromine anal fin blotches are phenotypically similar to the ones found in the genus *Callochromis* (Fig. 1). However, the phylogenetic position of *Callochromis*, which is nested within the Ectodini [25], suggests that these two types of blotches evolved independently. Overall, we envision two possible scenarios for the origin of egg-spots: in one case they represent a derived state of blotches found in haplochromines, whereas blotches found in ectodines evolved independently (two origins); alternatively egg-spots have evolved independently from the blotches of both basal haplochromines and ectodines (three origins).

Understanding the genetic pathways underlying these pigmentation phenotypes can help us to distinguish between these scenarios. While several studies have addressed pigmentation diversity in East African cichlids, little is known about the genetics underlying their coloration and pigmentation patterning, and only a handful of genes have been studied in detail. Among these genes is *hagoromo*, which shows a greater diversity of alternatively spliced variants and accelerated protein evolution in the haplochromines compared to other cichlids [26, 27]; *paired box 7* (pax7), on the other hand, was shown to be linked to a haplochromine female biased pigmentation phenotype [28]. Three genes have so far been associated with the egg-spot phenotype: the xanthophore marker colony stimulating factor 1 receptor A (*csf1ra*), and the two *four and a half lim domain 2* proteins (*fhl2a* and *fhl2b*). *csf1ra* is expressed in haplochromine egg-spots and in the characteristic “Perlfleckmuster” (pearly spotted) pattern present in cichlid fins. This gene underwent adaptive sequence evolution in the ancestral lineage of the haplochromines coinciding with the emergence of egg-spots [14]. However, *csf1ra* is downstream in the pathway of egg-spot morphogenesis. More recently, we have shown that *fhl2a* and *fhl2b* are more causally related to egg-spot development and that an alteration in the *cis*-regulatory region of *fhl2b* could have contributed to the emergence of this trait in haplochromines in the first place [15].

In this study, we first addressed the question of the genetic basis of the egg-spots. We then went onto use comparative transcriptomics across species carrying egg-spots and blotches to shed light on the origin of this novel trait. Specifically, we identified a total of 1229 genes that were differentially expressed (DE) between egg-spot and non-egg-spot fin tissues in the haplochromine cichlid *Astatotilapia burtoni*. These genes are evolving at a higher rate than average making this a valuable dataset to study the emergence and rapid diversification of this trait. For a subset of 46 DE genes we measured expression levels in three other species: the egg-spot bearing haplochromine *Cynotilapia pulpican*, carrying egg-spots on a different region of the anal fin than *A. burtoni*, and two blotch-bearing species, the basal haplochromine *Pseudocrenilabrus philander* and the ectodine *Callochromis macrops*. The rationale is that if egg-spots and blotches in haplochromines are controlled by the same genetic components they might show similar expression profiles.

A total of 29 out of 46 genes were found to be DE in *C. pulpican*. By comparing the expression in two haplochromine species with different egg-spot arrangements, we confirmed that the expression of the genes is correlated with the presence of egg-spots (irrespective of their position on the anal fin), whilst excluding potential positional genes and therefore confirming their involvement in egg-spots formation. Both types of blotches showed very distinct expression profiles from the egg-spots, and substantial differences in gene expression were also found between the two types of blotches. A similar gene expression profile between the egg-spots of derived haplochromines and the blotch pattern in the basal haplochromine *P. philander* would be indicative of a common origin for both traits, whereas similar expression profiles between the haplochromine egg-spots and the blotch of *C. macrops* would suggest that convergent evolution of this trait involved the same genetic pathways. Our study reveals the opposite for the genes under investigation, i.e. egg-spots and blotches show different expression profiles and also the two types of blotches differ in gene expression profiles, suggesting that egg-spots and blotches do not share a genetic basis and that convergent phenotypic evolution does not correspond to parallelism at the genetic level.

## Results and discussion

### Transcript profile in anal fin and egg-spot tissue

In order to identify genes involved in egg-spot morphogenesis we quantified differences in gene expression patterns between egg-spots and the surrounding non-pigmented anal fin of six *Astatotilapia burtoni* males (Fig. 1). Illumina RNAseq (RNA sequencing) provided a total of 193,054,988 high quality reads from the six egg-spot tissue samples and 194,099,061 reads from anal fin tissue samples of the same individuals. The replicates for each tissue were sequenced separately and the average number of reads per sample was 3,226,2837.42 (2,750,960.2–3,226,2837.42). We mapped the reads from each replicate to a reference *A. burtoni* embryonic library, which is a transcript collection from several different embryonic and larval developmental stages, and therefore probably the most comprehensive available representation of the entire gene set from *A. burtoni* [29]. In total we identified 1229 genes that were DE between the two types of tissues, with 620 genes being over-expressed in the egg-spot tissue, whilst 609 were under-expressed (Table 1). The DE transcripts, their identification using tBLASTx and BLASTx searches (against the NCBI non-redundant database [30]), together with the respective expression levels, are provided in Additional file 1. A first inspection of those DE genes between egg-spot and non-egg-spot tissue revealed that our experiment retrieved many genes with a known function in pigment formation and patterning in different model organisms including *paired box 7* (*pax7*), *endothelin receptor b1* (*ednrb1*), *microphthalmia*-*associated transcription factor a* (*mitfa*), *Agouti signaling protein 1* (*asip1*), *sex determining region Y box 10* (*sox10*) and *anaplastic lymphoma receptor tyrosine kinase* (*alk*) [31], suggesting that our strategy is a valid approach to identify candidate genes for egg-spot morphogenesis.

**Table 1:** Differential gene expression (DGE) statistics

DGE	Contigs	Contigs with BlastID	Annotated contigs
Over	620	377	178
Under	609	435	241
Total	1229	812	419

Number of genes over-expressed and under-expressed in the egg-spot, number of hits after BLASTx search against NCBI’s *Danio rerio* protein database and number of BLAST2GO annotated contigs

### Functional annotation of the DE genes

**Fig. 2:**
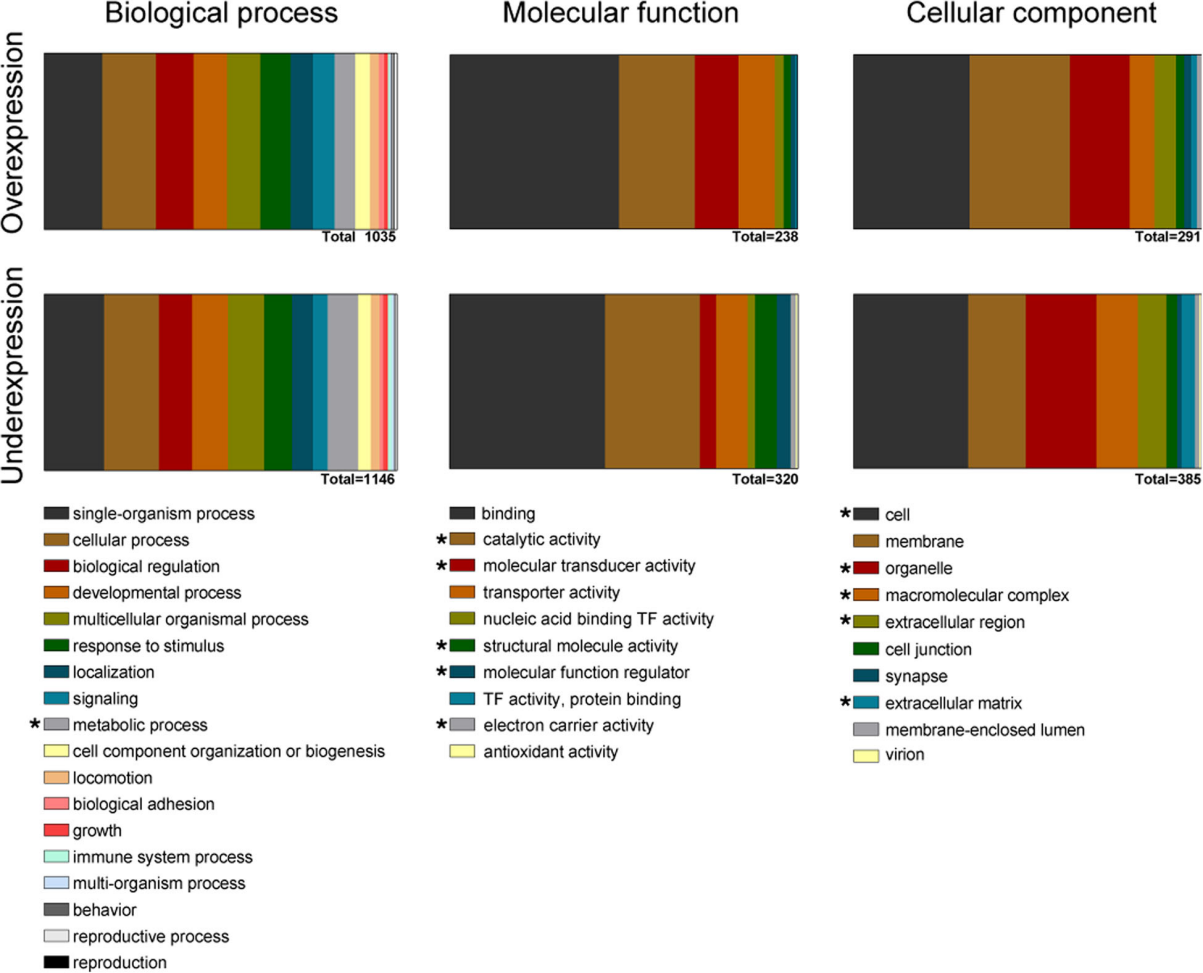
Gene ontology (GO) ID representations: (Biological process, Molecular function and Cellular component) for both over-expressed and under-expressed genes in the egg-spot tissue. Asterisks (in legend) denote significant differences in proportion of genes between the two datasets, as shown by chi-squared test (*p*-value < 0.05)

The reference *A. burtoni* transcriptome was annotated by performing a BLASTx search against NCBI’s *Danio rerio* protein database [30]. From the 1229 DE genes, 58.6 % (720) had significant BLAST hits against the database (annotated datasets can be found in Additional file 2), while 41.4 % (509) of the DE contigs were non-identified. From the 720 contigs with a BLAST hit we could functionally annotate 495 using BLAST2GO [32]. We further described the Gene Ontology (GO) term composition for egg-spot over-expression and egg-spot under-expression in comparison to the reference transcriptome GO representation (Fig. 2). Overall, the GO terms representation was similar between the two tissues. However, there were several GO terms for “Molecular function” and “Cellular component” that differed significantly between the two data-sets, suggesting, as expected, that the two tissues are functionally different (Fig. 2).

**Fig. 3:**
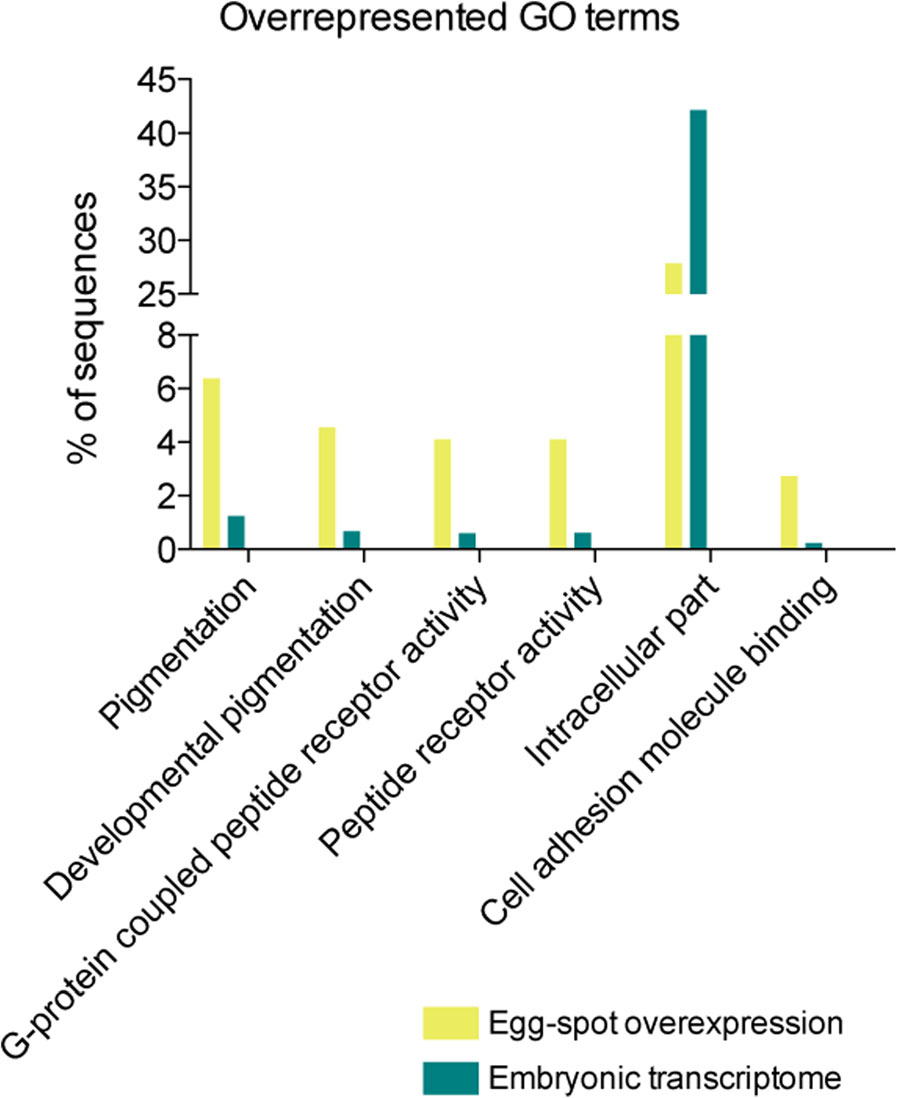
Enrichment of functional GO terms in the egg-spot over-expressed genes (yellow bar) when compared to the total transcriptome of *A. burtoni* (blue bar). Those were calculated with a two-tailed Fisher exact test (FDR < 0.05)

To narrow down the list of relevant GO terms, and to use them as a tool to find candidates, we used a two-sided Fisher’s exact test (false discovery rate (FDR) <0.05) to determine which functional GO categories were enriched in the genes over-expressed in the egg-spot in comparison to the total embryonic transcriptome. Five categories were significantly enriched in our over-expression gene dataset: ‘Pigmentation’ (GO:0043473), ‘Developmental pigmentation’ (GO:0048066), ‘G-protein coupled peptide receptor activity’ (GO:0008528), ‘Peptide receptor activity’ (GO:0001653) and ‘Cell adhesion molecule binding’ (GO:0050839) (Fig. 3). These are GO functional categories known to play a role in the development of pigmentation patterns. Neural crest cells are precursors of pigment cells and migrate from their original location to the anal fin where they will form the egg-spots [33–35], therefore genes playing a role in cell migration, cell adhesion and pigmentation development are relevant to the formation of this trait. Egg-spot formation relies on pigment production, which in turn is often activated via membrane receptor activity [36–38]. In Table 2 we present the list of genes belonging to these enriched functional categories that are potentially good candidates for egg-spot morphogenesis. The genes belonging to the GO term ‘Developmental pigmentation’ were overlapping with the ones included in the ‘Pigmentation’ category and the same is true for the two receptor GO term categories, therefore we only show three of the five enriched functional GO categories. This method of functional description of a gene dataset to extract candidates represents a supervised search, meaning that we might bias our findings towards what is already known. We note, however, that there are many other non-described genes, or known genes with incomplete GO term annotations, which could play a role in egg-spot morphogenesis.

**Table 2:** List of genes belonging to the GO term categories that are enriched in the egg-spot overexpressed dataset

	Gene	Transcript	logFC	BLASTx Identification	Accession	e-value
GO:0043473 Pigmentation						
1	ednrb	c5301_g0	0.926091617	endothelin B receptor [Haplochromis burtoni]	XP_005943243.1	0
2	rab38	c22025_g0	0.696999584	ras-related protein Rab-38 [Pundamilia nyererei]	XP_005720771.1	1.00E-149
3	pax7	c28600_g0	1.145533605	paired box protein Pax-7-like isoform X2 [Haplochromis burtoni]	XP_005948265.1	0
4	alk	c41674_g0	0.825942988	ALK tyrosine kinase receptor-like [Haplochromis burtoni]	XP_014192765.1	0
5	adrb1	c29399_g0	1.214559835	beta-1 adrenergic receptor [Pundamilia nyererei]	XP_005747452.1	0
6	gpnmb	c5056_g0	1.060188549	transmembrane glycoprotein NMB isoform X1 [Haplochromis burtoni]	XP_014191090.1	0
7	sox9a	c11994_g0	0.829590765	transcription factor Sox-9-A-like [Haplochromis burtoni]	XP_005923891.1	1.00E-127
8	mitf	c20716_g0	1.100168154	microphthalmia-associated transcription factor-like isoform X1 [Pundamilia nyererei]	XP_005731764.1	0
9	matp	c18656_g0	0.975922489	membrane-associated transporter protein [Haplochromis burtoni]	XP_005917392.1	0
GO:0001653 Peptide receptor activity						
1	ednrb	c5301_g0	0.926091617	endothelin B receptor [Haplochromis burtoni]	XP_005943243.1	0
2	calcrl	c8691_g0	1.204750964	calcitonin gene-related peptide type 1 receptor-like [Xiphophorus maculatus]	XP_005814950.1	2.00E-50
3	npyr1	c42378_g0	3.144400118	neuropeptide Y receptor type 1 [Haplochromis burtoni]	XP_005927047.1	0
4	rgr	c3216_g0	1.570045325	RPE-retinal G protein-coupled receptor [Haplochromis burtoni]	XP_005919610.1	3.00E-170
5	mc5r	c25961_g0	1.240861041	melanocortin receptor 5-like [Oreochromis niloticus]	XP_003452144.2	0
6	ackr3	c33293_g0	0.951452649	atypical chemokine receptor 3-like [Haplochromis burtoni]	XP_005950282.1	0
7	tacr3	c38449_g0	1.408219331	neuromedin-K receptor [Maylandia zebra]	XP_004549575.1	0
8	gcgr	c15641_g0	1.273842194	glucagon receptor [Haplochromis burtoni]	XP_005940348.1	0
GO:0050839 cell adhesion molecule binding						
1	jup	c20044_g0	0.785483788	junction plakoglobin-like [Haplochromis burtoni]	XP_014185585.1	0
2	postn	c318_g0	0.94955976	periostin-like isoform X2 [Haplochromis burtoni]	XP_005926524.1	0
3	cd200	c1300_g1	0.736112405	OX-2 membrane glycoprotein [Pundamilia nyererei]	XP_005747247.1	0
4	edil3	c4665_g0	1.050287092	EGF-like repeat and discoidin I-like domain-containing protein 3 isoform X1 [Oreochromis niloticus]	XP_005473287.1	0
5	cadm3	c4984_g1	0.941973544	cell adhesion molecule 3 isoform X1 [Haplochromis burtoni]	XP_005918142.1	0

### Potential lineage specific genes are DE in the egg-spot

How novel traits emerge and are modified is one of the many unresolved problems in evolutionary biology [39–41]. It has long been advocated that new traits can emerge via the co-option of conserved regulators [42]. More recently, however, evidence is accumulating that new, i.e. lineage specific, genes can also play an important role in the development of novel traits [43–45]. Around 41 % of our candidate contigs did not have a BLAST hit against the *D. rerio* protein database. This could be due to the incompleteness of this database or to the lack of homologs in this species. To control for these factors we performed BLASTx and tBLASTx searches against the NCBI non-redundant (nr) protein and nucleotide databases [30]. Around 15.5 % (191/1229) of the DE contigs could not be assigned to a specific gene present in either nr database (Additional file 1). The contigs without positive BLAST hits could represent non-coding RNAs, partial sequences of known genes that could not be identified, or lineage specific genes (new or fast evolving genes) [46]. These results add to previous work on comparative transcriptomics of East African cichlids reporting that only 51 % of the total transcriptomes of the species studied (*A. burtoni* and *Ophthalmotilapia ventralis*) have hits on the NCBI nr nucleotide database [46]. In our case, the reduction in percentage of non-identified contigs is, most probably, due to the recent availability of five cichlid genomes [29].

It has previously been shown that lineage specific genes might play a role in the emergence and development of novel traits. In cnidarians 15 % of the transcripts expressed in a phylum specific cell type are lineage-specific, though the functional role of these transcripts was not tested [45]. The relative contribution of novel genes to the evolution of new morphologies, when compared to the co-option of conserved genes, is still under debate and further studies are needed to clarify their role on the evolution of such traits. Therefore, it would be interesting to identify the unknown DE transcripts and assess their role in the development and evolution of egg-spots.

### Rates of evolution of the egg-spot DE genes

**Fig. 4:**
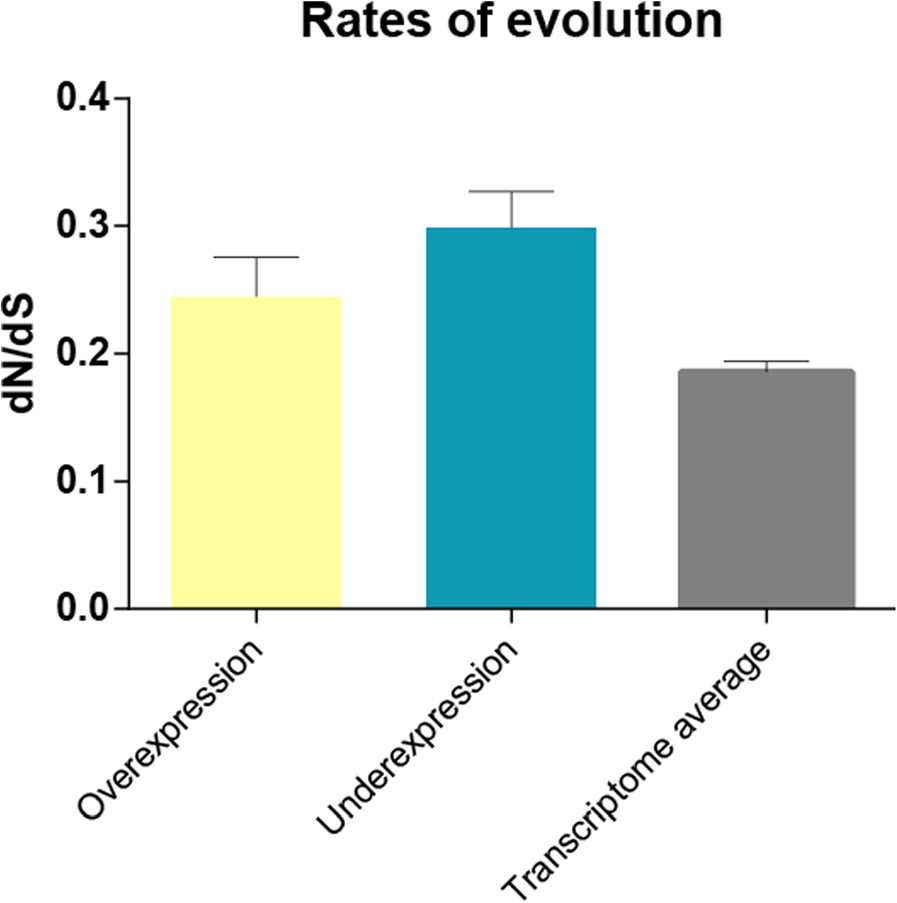
Rates of evolution (dN/dS) for the over-expressed genes (yellow bar), under-expressed genes (blue bar) and for a previously published dataset that estimated transcriptome-wide dN/dS values between cichlid species. No significant difference was detected between the over- and under-expressed dataset, although both had significantly higher dN/dS than the transcriptome average (as determined by *t*-test, *p*-value < 0.01). Error bars denote standard error of the mean

Changes in gene function can result either from modification in a *cis*-regulatory element that changes gene expression pattern and timing, and/or from a modification in the protein sequence that alters its function [47–50]. To test for protein sequence evolution in the egg-spot DE genes we calculated the rates of evolution in the form of dN/dS (ratio of non-synonymous substitutions over synonymous substitutions) of this gene dataset and compared the values obtained with a previously published dataset that estimated transcriptome-wide dN/dS values between cichlid species [46]. We were able to estimate dN/dS values (averages across species pair-wise dN/dS) for 196 out of the 1229 contigs (see Additional file 1). As expected, the majority of the genes were under purifying selection (dN/dS < 1) and there was no significant difference in the rates of evolution between the over and under-expressed genes (Fig. 4). However, for both the over- and under-expressed genes, the average dN/dS values were significantly higher than those of the entire transcriptome (Fisher’s exact test, *p*-value <0.05), which means that, on average, the genes that are DE between the egg-spot and the anal fin are evolving at a faster rate. The haplochromine egg-spot is a male ornamental trait and, hence, most likely under sexual selection, either directly via female choice or via male-male competition [17–21]. Our results thus provide support to the general finding that genes underlying sexually selected traits evolve more rapidly [51–54].

We found seven genes to be under positive selection (dN/dS > 1), four of which were over-expressed in the egg-spot tissue (Table 3). Among them there are genes that play a role in neural crest differentiation (*tenascin*) and in cell migration (*tenascin*, *mucin* and *family with sequence similarity 110c* (*fam110c*)), which are important processes in pigmentation development [55–58]. The other genes have no a priori functional link with egg-spot formation. Nonetheless, due to their difference in expression and their signature of adaptive sequence evolution, they should be considered as good candidates and their functional roles in egg-spot development should be tested in the future.

**Table 3:** DE genes under positive selection and their identification as determined through BLASTx against the NCBI non-redundant database

	Gene	Transcript	dN/dS	logFC	BLASTx Identification	Accession	e-value
1	FAM110C	c41094_g0	1.0613	−1.077081516	protein FAM110C [Haplochromis burtoni]	XP_005914672.1	6.00E-100
2	mucin-5 AC-like	c21845_g0	1.1477	1.528944965	mucin-5 AC-like [Haplochromis burtoni]	XP_005952554.2	0
3	intestinal mucin-like	c3522_g2	1.1479	0.741029968	intestinal mucin-like protein [Haplochromis burtoni]	XP_005941718.1	0
4	tenascin-like	c2897_g0	1.2524	2.61868262	tenascin-like [Haplochromis burtoni]	XP_005943223.1	0
5	myosin-IIIa	c23722_g0	1.2911	−0.911278787	myosin-IIIa isoform X5 [Haplochromis burtoni]	XP_014192226.1	0
6	polyubiquitin-like	c3172_g0	1.8501	−1.096942463	polyubiquitin-like [Haplochromis burtoni]	XP_014194859.1	1.00E-104
7	testican 1	c4037_g0	1.9352	1.068554755	testican-1 [Maylandia zebra]	XP_004545476.1	0

### Comparative gene expression via quantitative real time PCR

To confirm the results obtained via RNAseq, we examined a subset of 46 of the 1229 DE genes and tested their expression in egg-spot versus non-egg-spot tissue via quantitative real-time PCR (qPCR) in a second haplochromine species with a different egg-spot arrangement on the anal fin, *Cynotilapia pulpican* from Lake Malawi (Fig. 1). Half of these genes were over-expressed and half under-expressed in the egg-spot (Tables 4 and 5, respectively). These candidate genes were chosen randomly across the spectrum of the different levels of expression (from 1.3 to 5 fold differences in gene expression). Under-expressed genes were included as they might be acting as pigmentation inhibitors, thus preventing the appearance of egg-spots in other regions of the anal fin when over-expressed. Overall, there was no obvious trend with respect to functional GO categories associated with the top DE genes (see Additional file 2). Note that six out of the 46 candidates remained unidentified after tBLASTx searches against a non-redundant NCBI database.

**Table 4:** Differentially over-expressed transcripts and their identification as determined through BLASTx against the NCBI non-redundant database

	Gene	Transcript	logFC	BLASTx Identification	Accession	e-value
1	asip1	comp13033_c0	3.143700418	agouti-signaling protein-like [Oreochromis niloticus]	XP_003448419.1	3.00E-25
2	rbp7	comp8091_c0	3.229469794	retinoid-binding protein 7-like [Oreochromis niloticus]	XP_003448369.1	9.00E-91
3	hand2	comp22787_c0	3.511901296	heart- and neural crest derivatives-expressed protein 2-like [Oreochromis niloticus]	XP_003452793.1	2.00E-96
4	*NA*	comp17910_c0	2.484101474	No significant similarity found	*NA*	*NA*
5	*NA*	comp20229_c0	2.626648739	hypothetical protein LOC100708826 [Oreochromis niloticus]	XP_003455230.1	6.00E-19
6	IF ON3	comp1238_c0	2.271395094	intermediate filament protein ON3-like [Oreochromis niloticus]	XP_003441441.1	0
7	*NA*	comp23328_c0	2.751465615	No significant similarity found	*NA*	*NA*
8	akap12	comp28860_c0	2.392200617	A-kinase anchor protein 12 [Danio rerio] > gb|ABQ11279.1| gravin [Danio rerio]	NP_001091654.1	2.00E-49
9	bmp3b	comp14170_c0	1.907176985	bone morphogenetic protein 3B-like [Oreochromis niloticus]	XP_003438593.1	0
10	*NA*	comp23699_c0	2.056104188	No significant similarity found	*NA*	*NA*
11	rbp4a	comp104_c0	1.758056096	retinol-binding protein 4-A-like [Oreochromis niloticus]	XP_003441907.1	2.00E-132
12	hoxC12a	comp21426_c0	2.020913618	Hoxc12a [Haplochromis burtoni]	ABS70754.1	2.00E-172
13	cytl1	comp7733_c0	1.730109411	cytokine-like protein 1-like [Oreochromis niloticus]	XP_003441598.1	4.00E-80
14	*NA*	comp24816_c0	1.803818569	No significant similarity found	*NA*	*NA*
15	sfr5	comp6979_c0	1.70609137	secreted frizzled-related protein 5-like isoform 3 [Oreochromis niloticus]	XP_003451970.1	0
16	*NA*	comp4443_c1	1.661176264	No significant similarity found	*NA*	*NA*
17	fhl2a	comp2939_c0	1.543403442	four and a half LIM domains protein 2-like [Oreochromis niloticus]	XP_003453001.1	0
18	cecr5	comp6479_c0	1.505843782	cat eye syndrome critical region protein 5-like [Oreochromis niloticus]	XP_003457763.1	0
19	zygin1	comp2115_c0	1.527432266	fasciculation and elongation protein zeta-1-like [Oreochromis niloticus]	XP_003449843.1	0
20	vtn	comp7947_c0	1.490014821	vitronectin-like [Oreochromis niloticus]	XP_003458657.1	0.00E + 00
21	igf1	comp17864_c0	1.458424511	insulin-like growth factor 1 [Oreochromis niloticus]	XP_003448107.1	7.00E-94
22	igSF10	comp36206_c0	1.484184706	immunoglobulin superfamily member 10-like [Oreochromis niloticus]	XP_003454869.1	0
23	fmdo	comp19154_c0	1.343960756	fibromodulin-like [Oreochromis niloticus]	XP_003441412.1	0

While the egg-spots of *A. burtoni* are located in the proximal region of the anal fin, *C. pulpican* has its egg-spots in the distal region of the anal fin. By measuring the expression of these genes in this species, we effectively control for positional effects in gene expression along the proximal-distal axis.

We also aimed to determine whether egg-spots and blotches share a conserved gene expression profile, which would indicate a common origin of these two traits. We thus tested if the candidate genes identified in *A. burtoni* had similar expression levels in the blotches of a basal haplochromine species (*Pseudocrenilabrus philander*) and in the blotches of a member of a distinct cichlid tribe, an ectodine species (*Callochromis macrops*), where this trait has likely evolved independently.

### Comparative gene expression in haplochromine egg-spots

**Fig. 5:**
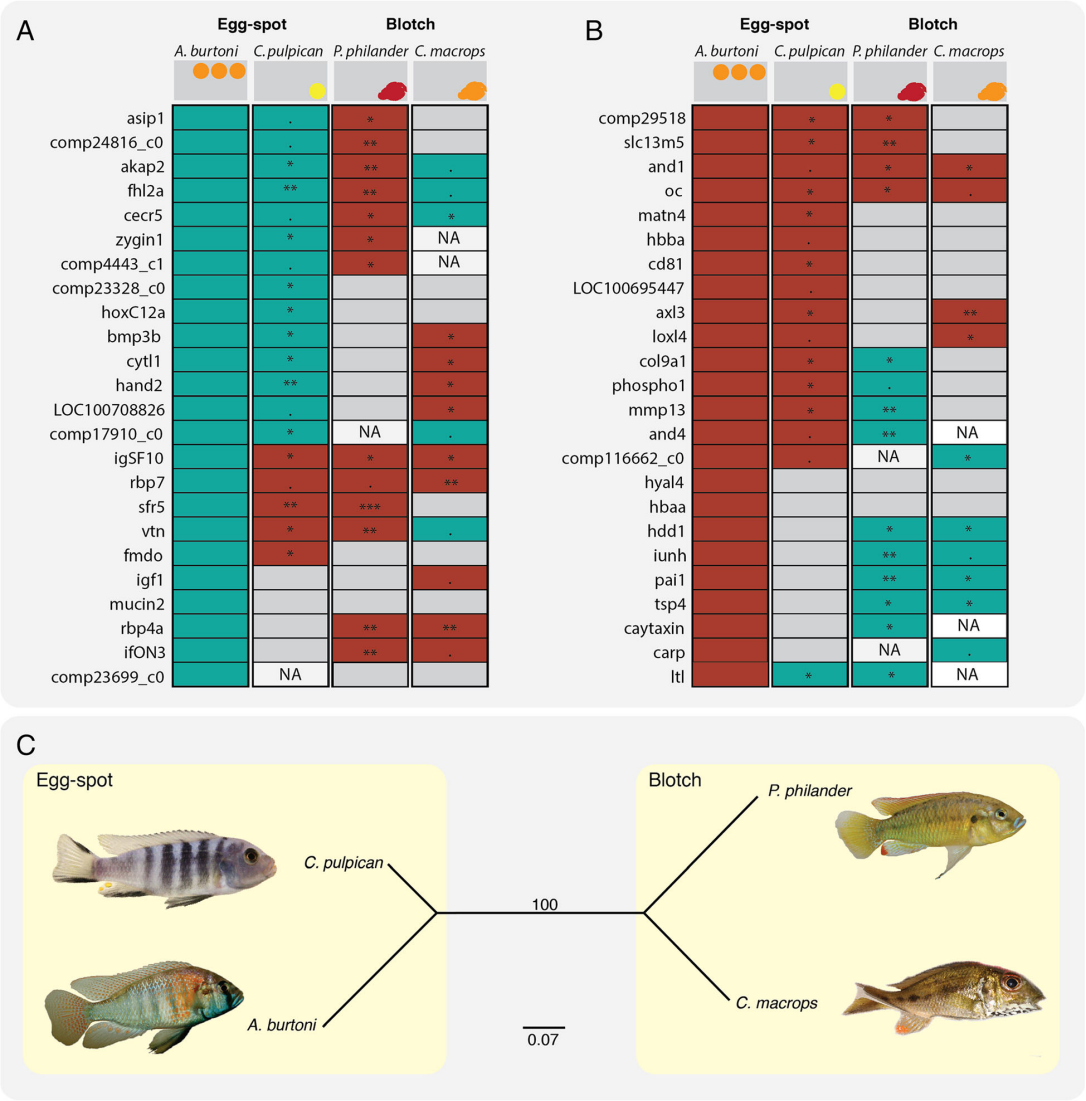
Gene expression results for 46 DE genes as measured by qPCR. qPCR was performed for *C. pulpican*, *P. philander* and *C. macrops* (Relative position of the egg-spot/blotch on the fin are shown on top of each panel). Expression of these genes was quantified in the egg-spots and blotches relative to the anal fin tissue. Blue box denotes over-expression, red denotes under-expression and grey denotes no significant difference. Instances where it was not possible to measure gene expression are colored white with NA. ***: *p* < 0.001, **: *p* < 0.01, *: *p* < 0.05, • *p* < 0.1 (for more details please see Additional files 4, 5 and 6). **a** Results for egg-spot over-expression dataset (Table 4). In the first column are the RNAseq results for *A. burtoni*. In the second, third and fourth column are the results for *C. pulpican*, *P. philander* and *C. macrops* respectively. **b** Results for egg-spot under-expression dataset (Table 5). Details of the statistical analyses used are found in Additional file 4 (*P.pulpican*), Additional file 5 (*P. philander*) and Additional file 6 (*C. macrops*) **c** Distance tree calculated using the gene expression results (over-expression, under-expression and no difference of expression) as characters

The qPCR gene expression analysis in the second haplochromine species revealed that 14 of the 23 genes that were over-expressed in the egg-spots of *A. burtoni* showed a similar expression pattern in *C. pulpican* (Fig. 5a), suggesting they are egg-spot specific and not simply involved in fin patterning. Among them are the previously identified egg-spot gene *fhl2a* [15], two transcription factors well known for their involvement in patterning and cell fate specification (*homeobox C12a* (*hoxC12a*) and *heart and neural crest derivatives expressed 2* (*hand2*)), and an important growth morphogen (*bone morphogenetic protein 3b* (*bmp3b*)) [59–61]. The detection of *fhl2a*, in particular, suggests that our results are robust, since the gene was recently shown to be over-expressed across egg-spot development [15]. Included in the list are five of the unidentified contigs.

The remaining nine genes that were over-expressed in the egg-spots of *A. burtoni* either showed no difference in expression (4) or were under-expressed (5) in the egg-spots of *C. pulpican* (Fig. 5a). These genes are most likely involved in fin rather than egg-spot patterning, as suggested by the fact that three of these of genes are known to participate in fin development (*retinol binding protein 7* (*rbp7*), *retinol binding protein 4* (*rbp4*) and *insulin*-*like growth factor 1* (*igf1*)) [62–64]. Overall, we confirmed the over-expression of 14 genes in the adult egg-spots from both *A. burtoni* and *C. pulpican* making them strong candidates genes for egg-spot formation that deserve further investigation.

Among the 23 under-expressed genes in *A. burtoni*, 15 were also consistently under-expressed in the egg-spots of *C. pulpican* (Fig. 5b), including one unidentified contig. Again, this suggests that these genes are egg-spot related. Among them is *aristaless 3* (*Axl3*), a gene belonging to the homeobox gene family, known for its patterning effects [65]. *Axl3* displays the highest expression differences among all genes (under- and over-expression included) and might putatively represent an inhibitor of the pigmentation/egg-spot pattern, although no role in pigmentation has been reported yet. The remaining eight genes showed no differences in gene expression between egg-spot and anal fin tissue on *C. pulpican*, and could therefore be involved in fin patterning. Thus far, none of these eight genes have been related to a function in pigmentation.

We cannot rule out that the genes that did not show the same pattern in both species do not have a function in egg-spots. Although egg-spots in *A. burtoni* and *C. pulpican* are homologous they do not necessarily have to share the exact same genetic network. It is thus possible that the DE genes might be responsible for interspecific differences of the egg-spot phenotype acting in a lineage-specific manner as has been shown in other taxa. For instance, the eyespots (concentric wing pigmentation patterns) of nymphalid butterflies, which are arranged along the distal half of the wing, are considered homologous [43, 66]. Nevertheless, there is a great flexibility in the expression patterns of four genes involved in the development of these structures in the different species studied: *antennapedia* was the only gene where there was a gain of expression associated with the origin of the eyespot phenotype, whereas there were many gain or loss events for *notch*, *distalless* and *spalt* in the different species [67]. Overall, the genetic network underlying the nymphalid eyespot pattern appears to be highly variable, suggesting that homologous structures are not necessarily controlled by the same set of genes. Perhaps the same is true for cichlid egg-spots, which might initially have been under the control of the same set of genes followed by diversification in the recruitment of different genes. A broader phylogenetic sampling of egg-spot phenotypes would be necessary to clarify this question.

The 29 genes that were consistently over- or under-expressed in the adult egg-spots in both haplochromine species are nevertheless strong candidates genes for egg-spot development and merit further investigation to understand their role in the origin and diversification of this trait. These genes should be studied in detail throughout development and their function should be tested, not only in one species but also across several species of egg-spot bearing haplochromines with variable egg-spot phenotypes. With this approach we will be able to distinguish between a functional role in the evolution of the trait and or merely a function in the development and/or physiology of the trait.

### Comparative gene expression between egg-spots and haplochromine blotches

We then measured gene expression of our set of 46 candidate genes in a basal haplochromine species, *Pseudocrenilabrus philander*, which displays a blotch rather than an egg-spot on its anal fin (Fig. 1). It is not known whether the blotches found in basal haplochromines are ancestral to the egg-spots found in ‘modern haplochomines’ [13, 25]. Homology inferences are typically made according to shared phenotypic criteria between traits and also according to parallelism at the developmental and genetic level [68] Therefore, if egg-spots and blotches are homologous we might expect that the gene expression patterns in both traits are, at least, partially conserved.

According to our results, haplochromine blotch and egg-spots differ substantially in their expression profiles (Fig. 5a, b). None of the 14 genes that were over-expressed in both *A. burtoni* and *C. pulpican* egg-spots were over-expressed in the blotch of *P. philander* (Fig. 5a), and only four of the 15 genes under-expressed in the two modern haplochromines were also under-expressed in *P. philander* (Fig. 5b). Although not conclusive, the poorly conserved expression pattern between the two traits suggests that the haplochromines’ egg-spots and the blotches have emerged independently within the Haplochromini lineage.

These results have to be taken with caution, though, as haplochromine egg-spots could have evolved from blotches by up-regulation of different effector genes within the same genetic network. This has been observed in *Drosophila*, where the phenotypically diverse wing pigmentation patterns are controlled by the key regulator *distalless* (*dll*) [49]. The emergence of this wing spot phenotype was brought by the evolution of regulatory links between *dll* and multiple downstream pigmentation genes, which resulted in their up-regulation in the wing [49].

### Comparative gene expression between eggs-spots, haplochromine and ectodine blotches

The blotch phenotype evolved more than once and is also found in some ectodine cichlids from Lake Tanganyika [12]. Ectodine anal fin blotches are similar to the ones found in basal haplochromines (Fig. 1), but apparently have an independent origin [25]. Although non-homologous, ectodine blotches might still share the same genetic network with both haplochromine egg-spots and blotches, as has previously been shown for other convergent traits [69].

In this study, we measured gene expression of our set of 46 candidate genes in the blotch of *Callochromis macrops*. Our gene expression assays revealed that only four of the genes that were over-expressed in *A. burtoni* and *C. pulpican* egg-spots were also over-expressed in the blotch of *C. macrops* (Fig. 5a). They encode transcription factors (*cat eye syndrome critical region 5* (*cecr5*)), co-factors (*fhl2a*) [70], cytoskeleton components and kinases (*a*-*kinase anchoring protein 2* (*akap2*)) and a non-identified transcript. These genes could be related to the pigmentation patterning or production of pigment in all three species. Furthermore, *C. macrops* also shares with *A. burtoni* and *C. pulpican* four genes that are consistently under-expressed in both species (Fig. 5b). One gene (*vitronectin* [71]) was over-expressed in *C. macrops* blotch and *A. burtoni* egg-spots, but not in *C. pulpican* egg-spots. These two species (*A. burtoni* and *C. macrops*) have in common that their egg-spots and blotches, respectively, contain orange pigments, while the egg-spot of *C. pulpican* is yellow. These genes might therefore correlate with patterning or production of orange pigment, although no such role has been previously described.

The comparison of expression profiles between the blotch bearing *P. philander* and *C. macrops* revealed that the underlying gene expression patterns are different indicating that there is probably no parallel evolution at the genetic level determining the phenotypic resemblance of the blotches. Curiously there are six genes that are under-expressed in the *A. burtoni* egg-spots that show no difference in expression in *C. pulpican*, but are over-expressed in blotches of both *P. philander* and *C. macrops*. The expression pattern of those six genes could be correlated to the blotch phenotype, but the most probable explanation is that they are involved in fin morphogenesis, since the non-pigmented region of *A. burtoni* matches the pigmented one in the two species with blotches.

### Gene expression clustering

To determine the relationship between the pigmented anal fin tissues (egg-spots and blotches), we coded the gene expression results of the 46 genes in the four different species into a matrix of discrete data points (0 – under-expression, 1 – no difference, 2 – over-expression) and constructed a distance genealogy (Fig. 5c). The resulting tree diagram shows a clear separation between egg-spot and blotch phenotype. The different species clearly cluster by gene expression phenotype (bootstrap of 100 %) and the observed similarities do not correspond to the species phylogeny (Fig. 5c, Table 6). The character distance matrix also shows that of the two blotches, *C. macrops* blotch is more similar to the haplochromine egg-spots in terms of gene expression (Fig. 5c, Table 6). Our results suggest that egg-spots, haplocromine blotches and ectodine blotches are not regulated by the same genetic components.

**Table 5:** Differentially under-expressed transcripts and their identification as determined through BLASTx against the NCBI non-redundant database

	Gene	Transcript	logFC	BLASTx Identification	Accession	E-value
1	axl3	comp20108_c0	−5.023523546	homeobox protein aristaless-like 3-like [Danio rerio]	XP_695330.1	2.00E-152
2	and1	comp5622_c0	−3.032229958	actinodin1 precursor [Danio rerio]	NP_001184183.1	4.00E-124
3	slc13m5	comp28513_c0	−3.143689749	solute carrier family 13, member 5 [Danio rerio]	NP_001136038.1	0
4	oc	comp5530_c0	−3.008253114	osteocalcin [Oreochromis niloticus]	XP_003443144.1	2.00E-62
5	*NA*	comp36289_c0	−3.547180945	hypothetical protein LOC100695447 [Oreochromis niloticus]	XP_003459280.1	2.00E-50
6	and4	comp2301_c0	−2.691704824	actinodin4 precursor [Danio rerio]	NP_001129716.1	1.00E-85
7	carp	comp10574_c0	−2.74115746	cocaine- and amphetamine-regulated transcript protein-like [Oreochromis niloticus]	XP_003456941.1	3.00E-58
8	*NA*	comp116662_c0	−2.679644343	No significant similarity found	*NA*	*NA*
9	*NA*	comp29518_c0	−2.574270324	No significant similarity found	*NA*	*NA*
10	hdd11	comp1748_c0	−2.191735413	putative defense protein Hdd11-like [Oreochromis niloticus]	XP_003446154.1	8.00E-127
11	iunh	comp29726_c0	−1.991962941	inosine-uridine preferring nucleoside hydrolase-like [Oreochromis niloticus]	XP_003455949.1	6.00E-55
12	hbba	comp70_c0	−1.747612794	hemoglobin subunit beta-A-like isoform 1 [Oreochromis niloticus]	XP_003442119.1	9.00E-99
13	matn4	comp4244_c0	−1.775099485	matrilin-4 [Oreochromis niloticus]	XP_003451941.1	0
14	tsp4	comp2186_c1	−1.66042901	thrombospondin-4-B-like [Oreochromis niloticus]	XP_003451568.1	0
15	mmp13	comp20376_c0	−1.855094613	collagenase 3-like [Oreochromis niloticus]	XP_003441718.1	0
16	col9a1	comp6219_c0	−1.614663219	collagen alpha-1(IX) chain-like, partial [Danio rerio]	XP_003200573.1	2.00E-138
17	caytaxin	comp7321_c0	−1.667845939	caytaxin-like [Oreochromis niloticus]	XP_003448582.1	0
18	ltl	comp656_c0	−1.547716431	lily-type lectin [Epinephelus coioides]	AEA39736.1	3.00E-69
19	phospho1	comp2411_c0	−1.545453078	probable phosphatase phospho1-like [Oreochromis niloticus]	XP_003442063.1	0
20	pai1	comp29400_c0	−1.616263913	plasminogen activator inhibitor 1-like [Oreochromis niloticus]	XP_003460165.1	0
21	hbaa	comp28_c0	−1.541854961	Hemoglobin subunit alpha-A	Q9PVM4.3	1.00E-79
22	loxl4	comp12727_c0	−1.470941331	lysyl oxidase homolog 4-like [Oreochromis niloticus]	XP_003455871.1	0.00E + 00
23	cd81	comp5209_c0	−1.491910445	CD81 antigen-like [Oreochromis niloticus]	XP_003443898.1	0.00E + 00

**Table 6:** Mean character distance matrix produced by PAUP

Species comparison		Distance	Genes that differ in expression
*P. pulpican*	*A. burtoni*	0.38297874	18
*P. philander*	*A. burtoni*	0.91111112	41
*P. philander*	*P. pulpican*	0.70454544	31
*C. macrops*	*A. burtoni*	0.79069769	34
*C. macrops*	*P. pulpican*	0.69047618	29
*C. macrops*	*P. philander*	0.47499999	19

Species cluster according to gene expression and not according to phylogeny

Overall our results suggest that haplochromine egg-spots, haplochromine blotches and ectodine blotches are novel pigmentation traits that evolved independently by re-using a limited number of common genes (Fig. 5 and Table 6). The genes in common seem to be related to the cellular composition of the trait, which is re-used every time a new pigmentation pattern emerges, and not with the pigmentation pattern per se. Therefore, a thorough comparison of the different fin phenotypes should be done to assess what are the cellular components of each of the pigmentation phenotypes to better understand and interpret the gene expression underlying it.

These homology inferences have to be taken with caution, as we have only studied a subset of candidate genes (46/1229) derived from the egg-spot versus non-egg-spot tissue transcriptomic comparison in *A. burtoni*. An in-depth comparison of the blotch tissue will certainly require comparative transcriptomics in the blotched species.

## Conclusions and future perspectives

Understanding the genetic and molecular basis of both evolutionary innovation and phenotypic variation is a major challenge in evolutionary biology. Using next-generation sequencing we here present a transcriptional survey of egg-spot tissue in the haplochromine cichlid *Astatotilapia burtoni*. This collection of DE transcripts represents the largest set of egg-spot candidate genes available and will greatly contribute to the understanding of the genetics underlying this trait. We provide a list of 1229 genes that are DE between egg-spots and non-egg-spots fin tissues, many of which are fast evolving genes that might be involved in the genetic network determining the egg-spot phenotype.

A closer look at the expression profiles of 46 of the DE genes shows that the expression profiles are not conserved between egg-spots and blotches, which suggests that haplochromine egg-spots, haplochromine blotches and ectodine blotches do not share the same genetic basis. This result indicates that these traits emerged independently in the evolution of this group of fishes. It has been hypothesized that egg-spots are modifications of the “Perlfleckmuster” (pearly spot) pattern that is present in fins of many cichlid species [12, 14]. In the future it will be interesting to determine if the same genes that underlie the egg-spots of haplochromines are also expressed in the “Perlfleckmuster”.

With our current approach, we identified 29 genes whose expression patterns are egg-spot specific in two distinct cichlid species, strongly pointing to a role in the formation of this trait. These genes definitely deserve further investigation; in particular, their expression dynamics should be examined during egg-spot development and their function should be assessed with transgenic experiments, now available for cichlids [72]. The functional characterization of these genes during egg-spot development and in a broader phylogenetic context will inform us about the origin and diversification of this innovation in the most species rich vertebrate lineage – the haplochromine cichlid fishes – thus leading to major advances in the understanding of the emergence and diversification of novel traits.

## Methods

### Samples

*Astatotilapia burtoni* and *Cynotilapia pulpican* bred laboratory strains were kept at the University of Basel (Switzerland) under standard conditions (12 h light/12 h dark; 26 °C, pH7). All individuals were euthanized with MS222 (Sigma-Aldrich, USA), following approved procedures (permit number 2317 issued by the Basel cantonal veterinary office) before tissue dissections. *Callochromis macrops* individuals were captured at Lake Tanganyika, Mpulungu (Zambia), *P. philander* were captured in a river near Mpulungu (both under a research permit issued by the Department of Fisheries, Republic of Zambia). Dissections were carried out *in situ*, tissues were stored in RNAlater (Ambion, USA) and shipped to the University of Basel.

### RNA extractions

Isolation of RNA was performed using TRIzol® (Invitrogen, USA). All dissected tissues were incubated in 750 μl of TRIzol and left at 4 °C overnight (or 8–16 hours). The tissues were homogenized with a BeadBeater (FastPrep-24; MP, Biomedicals, USA). Extractions proceeded according to manufacturer’s instructions and DNase treatment was performed with DNA-Free™ (Ambion, USA). RNA quantity and quality was determined with a NanoDrop 1000 spectrophotometer (Thermo Scientific, USA). cDNA was synthetized using the High Capacity RNA-to-cDNA kit (Applied Biosystems, USA).

### Differential gene expression analysis using RNAseq – Illumina

The anal fins of six *Astatotilapia burtoni* male juveniles were dissected and RNA was extracted from egg-spot and anal fin tissue for each individual. One microgram of RNA per sample was sent for library construction and Illumina sequencing at the Department of Biosystems Science and Engineering (D-BSSE), University of Basel and ETH Zurich. Samples were run in two lanes of an Illumina Genome Analyzer IIx (maximum read length was 50 base pairs (bp)).

The reads from each sample were mapped against a reference *A. burtoni* embryonic transcriptome that contains 171,136 reference transcripts. We mapped the reads from each library against the reference transcriptome using Bowtie2 as aligner [73] and RSEM (RNA-Seq by Expectation-Maximization) [74] as the method to estimate gene abundance. The individual RSEM files were concatenated into one single dataset and analyzed using the Bioconductor R package EdgeR [75]. Transcripts that had less than one count per million in one of the samples were discarded. We tested for differential expression between egg-spot and anal fin samples, using anal fin as reference. Since the samples were paired (each replicate of the egg-spot and anal fin belong to one individual fish), we included the individual information in the statistical model. For that we used a negative binomial generalized linear model (GLM) based on common dispersion using the individual as the blocking factor, i.e. we tested for consistent differences in expression between egg-spot and anal fin within individuals. Transcripts were considered as DE if, after correction for multiple testing, the false discovery rate (FDR) was lower than 0.05 [76].

### Functional annotation of differential expressed transcripts

Gene ontology (GO) [77] annotation of the differential expressed transcripts was conducted with Blast2GO version 2.5.0 [32]. BLASTx searches were done against the *Danio rerio* protein database using a threshold of *e*^−*5*^ and maximum number of hits of 20. These GO terms were used to estimate transcript function. A table with the list of the differential expressed transcripts, their respective values of expression, and their GO terms is provided in Additional file 1. Between dataset differences in the proportion of genes for individual level 2 GO terms were tested by means of chi-squared tests with *p*-values adjusted for multiple tests using Bonferroni corrections [78]. The enrichment of functional GO terms in the egg-spot over-expressed gene dataset was calculated with a two-sided Fisher’s exact test with a FDR of 0.05.

### Rates of evolution for the differential expressed transcripts

Transcriptome data from the five available cichlid species (*Pundamila nyererei*, *Neolamprologus brichardi*, *Oreochromis niloticus*, *Maylandia zebra*, and *Astatotilapia burtoni*) were downloaded from Broad Institute [29]. Each species’ transcriptome consisted of multiple libraries that were concatenated. The 1229 DE genes from *A. burtoni* were compared using a BLASTn search (threshold: *e*^−*50*^) against each species’ transcriptome and DE genes with a hit in all cichlid species were retained (599). The 599 DE genes were then compared using BLASTx (threshold: *e*^−*20*^) against the tilapia (*Oreochromis niloticus*) proteome from the ENSEMBL database and corresponding coding sequences (cds) retrieved (378). Finally, the database of 378 tilapia cds was queried against the individual cichlid transcriptomes using BLASTn (threshold: *e*^−*35*^). BLAST outputs were parsed and filtered to retain hits with identity >90 %, length >200 bp and bit score >200. We obtained 298 tilapia cds that have at least a hit on all cichlid transcriptomes. A concatenated fasta file was built to include the ten top hits from each cichlid transcriptome and the 298 tilapia cds. Sequences were then aligned using MAFFT v7.245 [79] with einsi –adjustdirection options (einsi is suitable for sequences containing large unalignable regions, as expected with the presence of UTRs (untranslated regions) and splicing variants in our transcriptome data). Alignments were trimmed using the tilapia cds as a reference and visually inspected. Alignments with paralogous sequences resulting from recent duplications were discarded. Within each individual alignment a consensus was built across transcripts from each cichlid species with ‘cons’ from EMBOSS [80] (−plurality 1.5, indicating the cut-off for the number of positive matches below which there is no consensus). Alignments were then translated to proteins and checked for all sequences being in the corresponding tilapia reading frame (no stop codons). The whole pipeline was run with customized R and Unix scripts. We obtained 196 good alignments, 74 % of which comprised of all five cichlid species sequences, while the remaining included at least three species each. Average alignment length was 1716 bp, ranging from 270 to 7794 bp. Alignments are available from the author upon request. dN/dS estimates were calculated using the script kaks.pl in Bioperl [81] which computes the dN/dS for all sequence pairs, using the Nei-Gojobori method [82].

### Gene expression analysis using qPCR

The expression of 46 genes (23 over-expressed genes in the egg-spot region and 23 under-expressed genes in the egg-spot) was further studied in three other species - *Cynotilapia pulpican*, *Pseudocrenilabrus philander* and *Callochromis macrops*. Primers were designed with GenScript Real-time PCR (TaqMan) Primer Design software available at https://www.genscript.com/ssl-bin/app/primer. Where possible, primers were designed in exon spanning regions to avoid effects of gDNA contamination. Primers were tested in all species and in cases where primers pairs did not work we designed new species-specific primers. Genes studied and primer sequences are available in Additional file 3.

Three qPCR experiments were carried out: *qPCR experiment 1*: Gene expression was compared between the non-egg-spot anal fin tissue and the egg-spot tissue of *C. pulpican*. This species has its egg-spot in a different position in the fin compared to *A. burtoni* (Fig. 1, *n* = 4–5). *qPCR experiment 2*: Gene expression was compared between the non-blotch anal fin tissue and blotch tissue of *P. philander* (Fig. 1, *n* = 6). In this experiment six individuals were used. *qPCR experiment 3*: Gene expression was compared between the non-blotch anal fin tissue and blotch tissue of *C. macrops* (Fig. 1, *n* = 4–7). In this experiment 4 to 7 individuals were used. For all experiments each individual was an independent replicate meaning that there was no pooling of samples.

The reactions were run on the StepOnePlus™ Real-Time PCR system (Applied Biosystems, USA) with FastStart Universal SYBR Green Master mix (Roche, Switzerland), following the manufacturer’s protocols. All reactions were performed with an annealing temperature of 58 °C, a final concentration of cDNA of 1 ng/μl and a final primer concentration of 200 ng/μl. The comparative threshold cycle (CT) method [83] was used to calculate the relative concentrations between tissues, where anal fin was taken as the reference tissue and Ribosomal protein L7 (*rpl7*) or the Ribosomal protein SA3 (*rpsa3*) genes as endogenous controls. Primer efficiencies were calculated using standard curves. Efficiency values of test primers were comparable to the efficiency of endogenous control primers (*rpl7*, *rspa3*) and are available in Additional file 3.

Significant differential gene expression between egg-spot/blotch and anal fin was tested with a paired *t*-test. When the data did not conform to the assumptions of a *t*-test (normal distribution and equal variances), an unpaired *t*-test with Welch’s correction or a Wilcoxon signed rank test was used. Normality of the data was tested using Shapiro-Wilk test and an *F*-test was used to determine if the variances of the datasets were equal. When the sample size was lower than five a Mann–Whitney test was used. Statistics were carried out using GraphPad Prism version 5.0a for Mac OS X (www.graphpad.com). Individual graphs for each gene studied and the details of the statistical results are given in Additional file 4 (*C. pulpican*), Additional file 5 (*P. philander*) and Additional file 6 (*C. macrops*). We could not test the expression of five of the genes for both datasets because the primers would not amplify at the required efficiency.

### Distance calculation and tree based on the genes expression results

The qPCR gene expression results were encoded into a matrix of discrete data points according to their expression level (0 – under-expression, 1 – no difference, 2 – over-expression). Consequently, a neighbor-joining distance tree based on this matrix was constructed using PAUP* 4.0b10 [84] with 100 bootstrap pseudoreplicates (Fig. 5c). To further test the hypothesis that the expression pattern corresponds to the phylogenetic signal, the mean character differences distance for all pairwise comparisons between species were calculated based on the matrix.

## Additional files

Additional file 1: Identity of the differential expressed genes between egg-spot and anal fin tissue together with the expression values and dN/dS estimations. (XLSX 222 kb) Additional file 2: Gene ontology annotation for the DE genes. (XLSX 148 kb) Additional file 3: Primers used in this study together with their efficiency values. (XLSX 50 kb) Additional file 4: qPCR results for *P. pulpican*. (*XLSX 257 kb*) Additional file 5: qPCR results for *P. philander*. (*XLSX 251 kb*) Additional file 6: qPCR results for *C. macrops*. (*XLSX 253 kb*)

## Abbreviations

Bp: Base pairs
CDS: Coding sequences
CT: Threshold cycle
DE: Differentially expressed
dN/dS: Ratio of non-synonymous substitutions over synonymous substitutions
FDR: False discovery rate
GLM: Generalized linear model
GO: Gene ontology
qPCR: Quantitative real-time polymerase chain reaction
RNAseq: RNA sequencing
RSEM: RNA-Seq by Expectation-Maximization
UTR: Untranslated region
